# Drive competition underlies effective allostatic orchestration

**DOI:** 10.3389/frobt.2022.1052998

**Published:** 2022-12-02

**Authors:** Oscar Guerrero Rosado, Adrian F. Amil, Ismael T. Freire, Paul F. M. J. Verschure

**Affiliations:** Donders Institute for Brain, Cognition and Behaviour, Radboud University, Nijmegen, Netherlands

**Keywords:** self-regulation, homeostasis, allostatic control, attractor model, need-based behavior, control theory

## Abstract

Living systems ensure their fitness by self-regulating. The optimal matching of their behavior to the opportunities and demands of the ever-changing natural environment is crucial for satisfying physiological and cognitive needs. Although homeostasis has explained how organisms maintain their internal states within a desirable range, the problem of orchestrating different homeostatic systems has not been fully explained yet. In the present paper, we argue that attractor dynamics emerge from the competitive relation of internal drives, resulting in the effective regulation of adaptive behaviors. To test this hypothesis, we develop a biologically-grounded attractor model of allostatic orchestration that is embedded into a synthetic agent. Results show that the resultant neural mass model allows the agent to reproduce the navigational patterns of a rodent in an open field. Moreover, when exploring the robustness of our model in a dynamically changing environment, the synthetic agent pursues the stability of the self, being its internal states dependent on environmental opportunities to satisfy its needs. Finally, we elaborate on the benefits of resetting the model’s dynamics after drive-completion behaviors. Altogether, our studies suggest that the neural mass allostatic model adequately reproduces self-regulatory dynamics while overcoming the limitations of previous models.

## Introduction

As 19th-century physiologists Claude Bernard and Ivan Pavlov proposed, living systems are generally characterized by their ability to self-regulate (Bernard, 1865; Pavlov, 1955). Through self-regulation, an organism ensures its fitness by adjusting its inner processes relative to external perturbations (Papies and Aarts, 2016), in turn, assisting self-maintenance (i.e., Autopoiesis) (Maturana and Varela, 1991). One aspect of self-regulation is homeostasis, which describes the process of maintaining an internal state within a desirable range as proposed by Cannon (Cannon, 1939). Once external perturbations produce a deviation from the desirable range, a homeostatic error arises, driving a proportional error-correcting response to restore balance in the system. This process would be later formalized as a feedback control loop by Norbert Wiener (Wiener, 1948), father of cybernetics. However, the homeostatic control of a single need is insufficient to ensure fitness since living organisms need to maintain a rather large set of internal needs with dynamically varying priorities from electrolysis to temperature and oxygenation. To overcome this and other gaps of homeostasis in explaining self-regulation, the notion of allostasis aims to capture the stability of the integrated self rather than its parts.

Allostasis transcends the constancy imposed by homeostasis by aligning all organism’s internal parameters with the environmental demands and opportunities (Sterling, 1988). In its broad conceptualization, allostasis targets stability by integrating many dynamic regulatory principles (Sterling, 2020). For this reason, we differentiate three complementary and coupled levels of allostasis. First, allostatic orchestration allows the agent to rapidly rank its internal needs based on priorities, urgencies, and opportunities. Second, predictive allostasis leverages environmental regularities to learn associations between external events and internal states, supporting anticipatory allostatic orchestration and future homeostatic risks. Finally, contextual allostasis benefits from goal-oriented learning mechanisms to enrich self-regulatory strategies with spatio-temporal information, in turn facilitating anticipatory behavioral strategies. Hence, we consider that in the mammalian brain allostatic regulation of action is organized in a multi-layered architecture following the Distributed Adaptive Control (DAC) theory (Verschure, 2012, 2016).

While nowadays, allostasis is increasing in popularity (2010: 2,584 citations *versus* 2021:12549 citations, resource: Scopus), most of the latest computational models on allostasis focus on exploring the advantages of its predictive component (Sterling, 2012). Paradoxically, some models return to the one-single-need problem of homeostasis (Tschantz et al., 2022). In addition, the concept of allostasis has not explained the computational mechanisms by which individuals achieve stability by orchestrating different homeostatic systems. The divergent modeling approaches adopted by the few computational studies addressing allostasis demonstrate the lack of consensus when determining the fundamental principles behind allostatic orchestration.

In 2010, Sanchez-Fibla et al. developed what, to our knowledge, is the first computational model of allostatic orchestration (Sanchez-Fibla et al., 2010). The model emulated rodent behavior and physiological states in an open field test (Gould et al., 2009) with simulated and physical robots. This model proposed that the animal’s behavior resulted from the interaction between two internal needs: Security, which is fulfilled in one of the arena’s corners representing the rodent’s home base, and arousal, which would be higher in the center of the arena given the maximum exposure of the animal at that location. However, although reproducing the animal’s overall trajectory pattern and occupancy preferences, the model did not elaborate on the neuroscience supporting allostasis.

A more recent model bases the optimal selection of regulatory behaviors on maximizing a subsequent reward (Laurençon et al., 2021). This deployment of a reward-based allostatic model builds on the premises of homeostatic reinforcement learning (HRL) (Keramati and Gutkin, 2014). HRL represents a major refinement of traditional reinforcement learning theories grounding learning protocols on the individual’s internal state. HRL successfully explains effects in animal behavior such as alliesthesia, namely, the fluctuations in reward value during resource acquisition (Cabanac, 1971). Still, although HRL leverages temporal and spatial information to improve the self-regulatory strategy, this approach makes a complementary learning process critical for solving the allostatic orchestration problem.

Finally, a third approach suggests that allostatic orchestration emerges from motivational conflict solved *via* attractor dynamics (Jimenez-Rodriguez et al., 2020).In this approach, the attractor dynamics implement competition through cross-inhibition (Usher and McClelland, 2001; Marshall et al., 2015). This framework supports the idea that attractor dynamics can underlie the optimal selection of regulatory behavior and explained their duration and latency. However, an explanation of how neural correlates of internal needs’ implement such competing dynamics is unclear.

Grounding the design principles of a model of allostasis in state-of-the-art neuroscience constitutes a pending task for previous modeling approaches that have largely relied on algorithmic solutions. We suggest that novel approaches can overcome this challenge by focusing on the core behavior systems of the mammalian brain (Merker, 2013; Verschure, 2016). Interoception of physiological needs such as hydration, nutrition, thermoregulation, or sleep is generally attributed to distinct specialized hypothalamic nuclei (Strecker et al., 2002; Blouet and Schwartz, 2010; Nakamura, 2011; Zimmerman et al., 2017). In contrast, other psychological needs (e.g., social interaction) depend on more distributed brain networks (Lee et al., 2021). Importantly, recent studies suggest that these nuclei, and so the internal needs they represent, are not independent of each other but hold a competing relationship through inhibitory interactions (Burnett et al., 2016; Osterhout et al., 2022; Qian et al., 2022). This competition between internal drives could serve as the basis of allostatic orchestration by imposing a winner-take-all mechanism represented as attractor dynamics. Thus, irrelevant drives are suppressed, and the singleness principle of action is supported (Sherrington, 1906). This research literature suggests that an attractor-based approach is suitable for modeling allostasis.

In contexts where animals constantly self-regulate multiple internal needs, decision-making could be hampered by attractor forces sustained after drive-completion behaviors. Indeed, *in vivo* studies suggest that cortical areas involved in decision-making operate in a critical regime (close to a phase transition) (Ma et al., 2019) that occasionally evolves to supercriticality (saturated population response), a regime that supports effective information transmission (Li et al., 2019). Nonetheless, how the system returns to criticality from a supercritical regime is poorly understood. The paraventricular hypothalamic nucleus (PVH) is a good candidate to inhibit the main interoceptive nuclei, recover their basal population activity after drive-completion behaviors, and create the initial conditions for the next cycle of allostatic orchestration. From one side, PVH mediates many diverse motivational functions, including thirst (Zimmerman et al., 2017), hunger (Blouet and Schwartz, 2010), and thermoregulation (Nakamura, 2011). Conversely, corticotrophin-releasing hormone (CRH) neurons in the PVH are suggested to be sensitive toward reward acquisition. Specifically, PVH CRH neurons get inhibited during drive-completion behaviors, representing a potential source of global inhibition to the rest of hypothalamic interoceptive nuclei (Yuan et al., 2019). In this research work, we model this form of decision reset by applying general inhibitory inputs to the excitatory populations once an internal need has been satisfied.

In the following sections, we will describe a novel neural mass model of allostatic orchestration grounded on the interoceptive mechanisms of the mammalian brain. As in (Sanchez-Fibla et al., 2010), the model will be embedded in an agent endowed with a so-called core behavior system (CBS) (Merker, 2013; Verschure, 2016). In other words, besides sensing its internal environment, a competence attributed to the hypothalamus (Strecker et al., 2002; Blouet and Schwartz, 2010; Nakamura, 2011; Zimmerman et al., 2017), the agent can orientate in an external environment and perform basic navigation based on an appetitive-aversive axis, cognitive functions attributed to the superior colliculus and the zona incerta-periaqueductal gray axis, respectively. The resultant model is tested in both static and dynamic environments. In the static condition, we aim to elucidate if our model can faithfully replicate both previous models of allostatic control and rodent behavior in an open field test (Gould et al., 2009). In the dynamic environment, we will further explore the robustness of our model when environmental opportunities to satisfy internal needs decrease over time. Finally, we will analyze how inducing subcritical dynamics after drive fulfillment facilitates switches in self-regulatory strategies.

## Materials and methods

**FIGURE 1:**
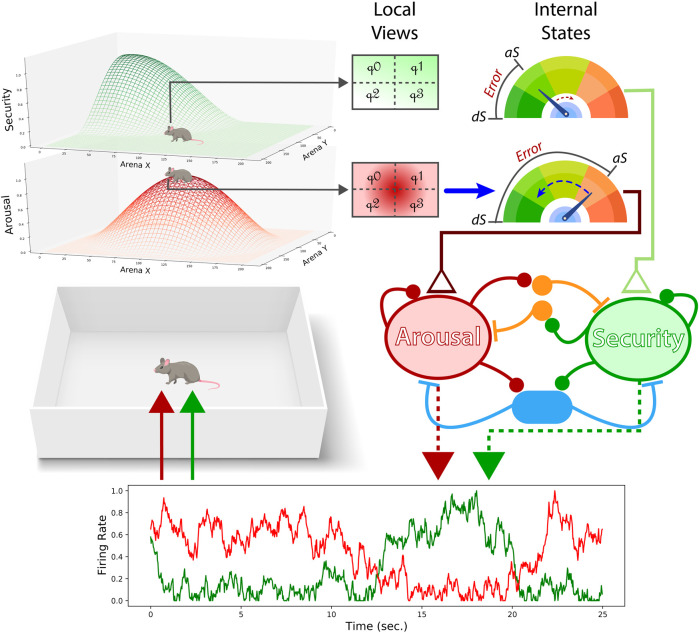
Performance of the neural mass allostatic model in an open field. Two gradients represent the areas where the two internal needs, arousal (red) and security (green), can be fulfilled (top-left). The agent partially observes those gradients through local sensation (top-middle). Local sensation allows the agent to adjust its actions to ascend/descend the gradients and detect when the observer’s current position is in the vicinity of the resource area (i.e., the peak of the gradient). If the agent is not close enough to the resource area, the internal state related to that resource keeps declining, as the security actual state (aS) is doing in this figure (top-right). In contrast, if the agent occupies the peak of the gradient, the internal state approximates the desired state (dS), as shown with arousal. The aSs and dSs are compared, creating a homeostatic error (top-right) that will input their respective excitatory pools in the neural mass model (middle-right). The level of competition is defined by the ratio of mutual inhibition (orange interneurons) and shared feedback inhibition (blue interneurons). Finally, the firing rate of each excitatory population provides the agent with the corresponding drives given its internal states.

To better understand the potential of competing dynamics between internal drives in facilitating need orchestration and stability of the self, we built a novel allostatic model grounding its design on contemporary research literature. Consequently, a neural mass model was built incorporating two distinct populations sensitive to homeostatic markers while holding a competing relationship (Figure 1). Aiming for convergent validation, we equipped a synthetic agent with this biologically-constraint model and analyzed its ability to defend its internal states by navigating an external environment.

### Homeostatic systems

Assuming that living organisms have interoceptive capabilities to assess their internal state, we conceptualized each internal need as a homeostatic mechanism. Here, actual and desired states are compared providing a measure of homeostatic error (that is, *hEi* = |*dSi* − *aSi*|). Importantly, homeostatic systems have no power to provide regulatory responses by themselves. Instead, they bias those behaviors by constantly feeding the neural mass allostatic model with homeostatic errors (Figure 1).

The transition between actual internal states responded to the following dynamical law,

$$aS_{i}\left(t\right)=aS_{i}\left(t-1\right)-dR_{i}\left(t\right)+rI_{i}\left(t\right)$$ (1)

where *aS*_*i*_(*t* − 1) is the actual state of the homeostatic system *i* in the immediate previous timestep, *dR*_*i*_(*t*) corresponds to the decay rate applied to each homeostatic system at time *t*, and *rI*_*i*_(*t*) represents the resource impact at time *t*. Importantly, resource impact will be 0 unless the agent is in the vicinity of the resource. By applying different decay factors and resource impacts, internal states can evolve with different temporal dynamics.

This dynamical law, similarly applied in (Jimenez-Rodriguez et al., 2020), notably differs from the methods applied in (Sanchez-Fibla et al., 2010), where the agent drew its internal states directly from gradients mapped onto the two-dimensional arena.

### The neural mass allostatic model

Wilson-Cowan equations modified as in (Amil and Verschure, 2021) were used to model two need-sensitive neural populations (Figure 1). This modification allowed to account for mutual and shared feedback inhibition held between the excitatory populations, as follows:

$$\tau \frac{dD_{1}\left(t\right)}{dt}=-D_{1}+f\left(w_{+}D_{1}+hE_{1}-Qw_{-}D_{2}-\left(1-Q\right)w_{=}f\left(D_{1}+D_{2}\right)\right)+\sigma \xi \left(t\right)$$ (2)

$$\tau \frac{dD_{2}\left(t\right)}{dt}=-D_{2}+f\left(w_{+}D_{2}+hE_{2}-Qw_{-}D_{1}-\left(1-Q\right)w_{=}f\left(D_{1}+D_{2}\right)\right)+\sigma \xi \left(t\right)$$ (3)

where *f*(*x*) is the logistic *f* − *hE*_*i*_ function,

$$f\left(x\right)=\frac{F_{\max}}{1+e\frac{-\left(x-\theta \right)}{k}}$$ (4)

In these equations, *τ* is the time constant determining the timescale of population dynamics. *D*_*i*_ is the drive magnitude represented as the mean firing rate of the excitatory population *i*. *w*_+_, *w*_−_, and *w*_=_ are the weights for recurrent connections within the excitatory population, mutual inhibition, and feedback inhibition, respectively. *Q* represents the mutual/feedback inhibition ratio, a variable that allows for inducing a controlled level of competition. *σ* and *ξ* are the variance and magnitude of Gaussian noise provided to the excitatory populations (See Supplementary Figures S1–S4 for *Q* and noise parameter search). Finally, *F*_max_, *k*, and *θ* are the maximum firing rate, gain, and threshold parameters of the *f* − *hE*_*i*_ logistic curve respectively (Eq. 4).

### Orientation

Methods enabling the orientation and navigation of the synthetic agent in the arena are based on (Sanchez-Fibla et al., 2010). In both conditions (static and dynamic environments), we served from three-dimensional gradients to represent the location of resources fulfilling specific internal needs. Notably, gradients are used solely to support navigation, and internal states are not directly linked to the agent’s location in the arena, as in the original study. Instead, internal states follow their dynamics, as explained above. The decision to use gradient-based methods for navigation is supported by representations of future navigational goals in the orbitofrontal cortex (Basu et al., 2021).

We implemented a partially observable environment by providing the agent with local sensations of the gradient areas surrounding its position. The local sensation was divided into four quadrants (Figure 1) to allow goal-directed-like navigation. By observing differences between the upper horizontal quadrants ( $q_{i}^{0}$ and $q_{i}^{1}$ ), $H_{i}^{\mathrm{sign}}$ controlled the agent’s orientation. $AD_{i}^{\mathrm{sign}}$ implements an appetitive-aversive behavioral axis (gradient ascent/descent) by comparing agent (*aGL*) and resource gradient location (*rGL*); understanding *aGL* as the mean gradient value between the four quadrants and *rGL* as the gradient value at its peak.

$$H_{i}^{\mathrm{sign}}=\left\{\begin{array}{cc} 1\; & if\;q_{i}^{0}<q_{i}^{1}-th\\ -1\; & if\;q_{i}^{0}>q_{i}^{1}+th\\ 0\; & \text{otherwise} \end{array}\right.$$ (5)

$$AD_{i}^{\mathrm{sign}}=\left\{\begin{array}{cc} 1\; & if\;aGL_{i}^{0}<rGL_{i}^{1}-th\\ -1\; & if\;aGL_{i}^{0}>rGL_{i}^{1}+th\\ 0\; & \text{otherwise} \end{array}\right.$$ (6)

### Internally-driven navigation

Outputs of the excitatory populations of the neural mass allostatic model are integrated with $H_{i}^{\mathrm{sign}}$ and $AD_{i}^{\mathrm{sign}}$ orientational signals to result in internally-driven goal-oriented-like navigation, following:

$$Navigation=c+\left(\overset{\mathrm{nGrad}}{\underset{i=1}{\sum }}H_{i}^{\mathrm{sign}}\cdot AD_{i}^{\mathrm{sign}}\cdot Exc_{i}^{\mathrm{output}}\right)\cdot \frac{1}{nNeeds}$$ (7)

where *c* is a constant ensuring a default action going forward, $Exc_{i}^{\mathrm{output}}$ accounts for the output of the excitatory population *i* of the neural mass allostatic model, and $\frac{1}{nNeeds}$ is a normalization factor given the number of implemented needs.

### Experimental design

A first study was conducted to evaluate the competence of the neural mass allostatic model in replicating rodent behavior during an open field test. In this study, a synthetic agent navigated a static simulated environment to defend its internal states of security and arousal. 50 experiments were carried out to analyze navigational patterns consistency and the internal dynamics of the agent along the simulation.

In the second study, the synthetic agent endowed with the neural mass allostatic model incorporated two distinct internal needs: thermoregulation and hydration. A dynamic simulated environment allowed us to assess the agent’s ability to adapt its navigation according to environmental opportunities. 50 experiments were carried out to analyze the consistency of the navigational patterns, the internal dynamics taking place along the simulation, and the relationship between these internal dynamics and environmental changes.

In both the first and the second studies, the agent must constantly decide what internal drives should base its navigation on to maximize stability. This continuous decision-making condition represents a major difference from the original work in which the model’s design is based on (Amil and Verschure, 2021) and is a novel challenge to overcome. Therefore, a third study was conducted to evaluate the advantages of inducting subcritical dynamics after need resolution. As in the second study, a synthetic agent navigated a dynamic environment to defend its internal states of hydration and thermoregulation. 50 experiments were conducted to assess the advantages of applying global inhibition after drive-completion behavior compared with the second study, where we did not consider such an effect.

### Arenas

The arenas simulated in static and dynamic conditions were designed as two-dimensional 200 × 200 matrices incorporating one gradient per internal need. These gradients represented the location in the two-dimensional space where each internal need can be satisfied.

**FIGURE 2:**
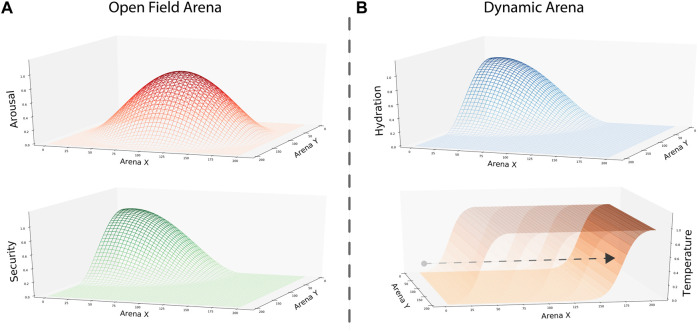
Gradients representing environmental opportunities to satisfy internal needs in each condition. **(A)** Arousal and security gradients were designed to replicate rodent behavior in an open field test. To represent the maximal level of exposure when exploring the center of the arena, we set the peak of the gradient in that location. Similarly, we used the peak of the security gradient to represent the home base location in one of the corners. **(B)** Hydration and temperature gradients were designed to test the performance of our model in a dynamic setting. Here, the hydration gradient was static, with its peak in one of the corners. In contrast, the temperature gradient changed over time. To do so, we built the gradient as a two-dimensional sigmoidal function where its *x*-intercept increased as the simulation evolved. Thus, the peak area where internal temperature increases shrunk, and the intermediate area between gradients increased over time.

In the first study, we built a static environment following the gradient design of the open field test used in (Sanchez-Fibla et al., 2010). Thus, we implemented two gradients mapping the opportunities to calm security and arousal drives. The security gradient was designed as a Gaussian gradient with its peak in the top-left corner, representing the home base location. Meanwhile, the arousal gradient was designed with its peak in the center of the arena, representing the animal’s exposure level. The configuration of these gradients aimed to replicate rodent navigational patterns, understood as a preference to occupy the home base and to explore the arena close to the walls (i.e., thigmotaxis) with occasional transversals (Figure 2A).

In the second and third studies, a dynamic environment implemented two gradients to map the opportunities to satisfy thermoregulation and hydration internal needs. We used a similar static gradient to security in the first study to represent the thirst-calming resource—nonetheless, the gradient representing the area where internal temperature increases changed over time. Designed as a two-dimensional sigmoidal function, the area providing temperature was large at the beginning of the simulation. However, as the simulation evolved, the *x*-intercept of our sigmoidal gradient increased, shortening the temperature gradient’s peak area and establishing a larger gap between the two resource locations (Figure 2B). Thus, at the experiment’s end, the temperature gradient’s peak area only covers a short area of the arena at its bottom limit.

### Efficiency, fairness and stability

To further understand the relationship between internal and external variables, in the second and third studies, we compute three measures traditionally used in game theory (Binmore, 2005; Hawkins and Goldstone, 2016; Freire et al., 2020). Efficiency provides a measure of how good the system is in maintaining the internal states close to their setpoints. Fairness designates whether there is any bias in the engagement of drives. Lastly, stability, computed as the mean square homeostatic error, comprises both the magnitude and the difference between homeostatic errors to provide a general measure of the agent performance.

To inform about the evolution of these measures throughout the experiment, they were calculated for every 1/100 faction of the duration of the experiment. Hence, mean internal and desired states only represented those elements in the considered fraction across the 50 experiments.

$$Efficiency=\frac{meanT+meanH}{nNeeds}$$ (8)

$$Fairness=\frac{\left|meanT-meanH\right|}{nNeeds}$$ (9)

$$Stability=1-\overset{\mathrm{nGrad}}{\underset{i=1}{\sum }}{\left(meanIS_{i}-meanDS_{i}\right)}^{2}$$ (10)

## Results

### Open field test

**FIGURE 3:**
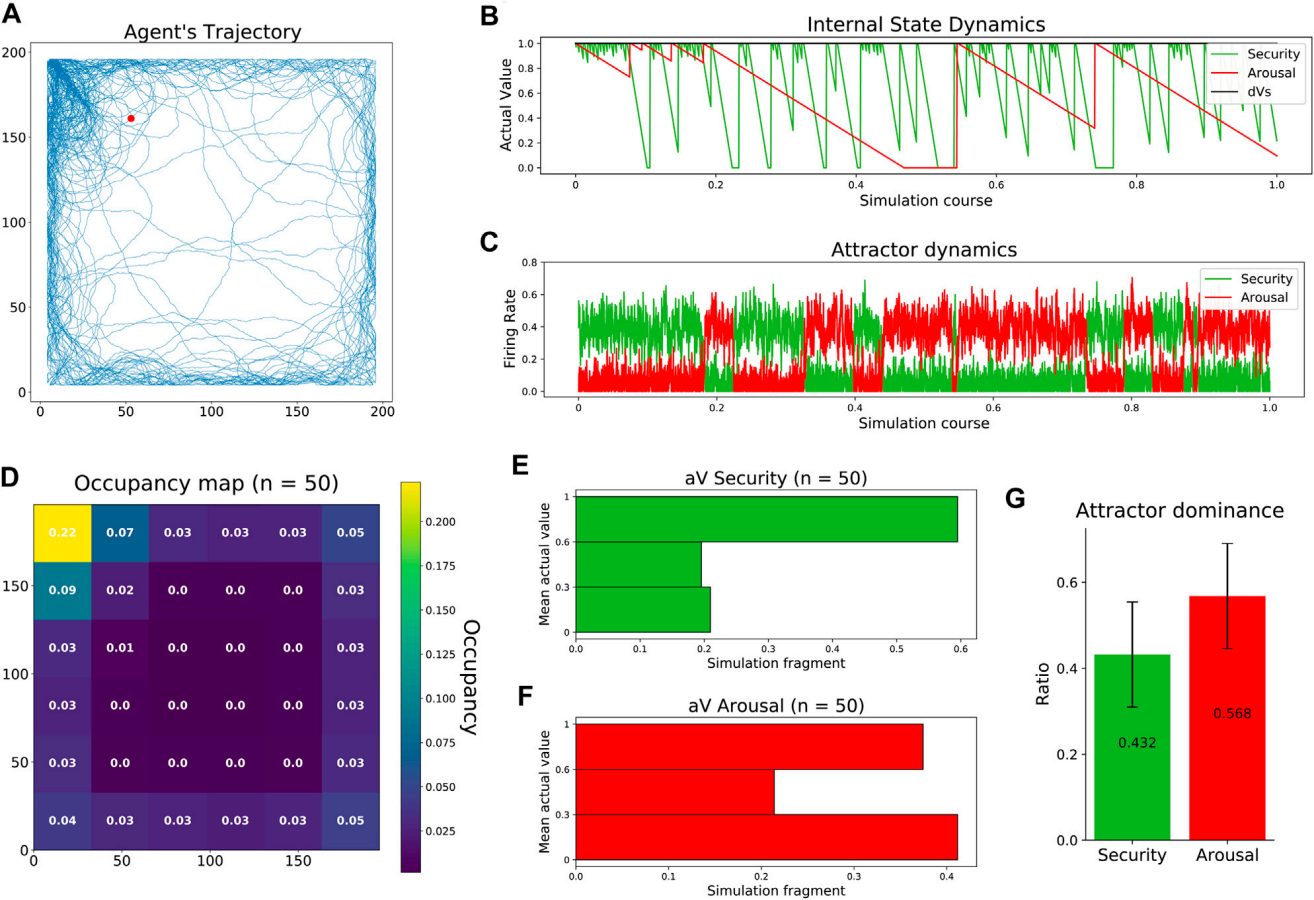
Replication of rodent behavior in an open field test. **(A)** Agent’s trajectory tracked along a complete experiment. The red dot represents starting location, randomized across experiments. **(B)** Agent’s internal state dynamics during a complete experiment (same as a). **(C)** Firing rates of the excitatory populations configuring the neural mass allostatic model during a complete experiment (same as a). **(D)** Occupancy map across 50 experiments with normalized values. **(E)** Distribution of security states across 50 experiments. **(F)** Distribution of arousal states across 50 experiments. **(G)** Mean attractor dominance across 50 experiments.

In the first study, as previous works did (Sanchez-Fibla et al., 2010), we aim to reproduce the navigational patterns of a rodent in an open field test. As in rodents, the trajectories of our agent showed preferences toward the walls (thigmotaxis) and the top-left corner (home base). At the same time, occasional transversals explored the center of the arena (Figure 3A, Supplementary Figure S5). The observed trajectory patterns resulted from the competition between two drives with different temporal dynamics. Fast-decaying security made the agent constantly revisit its home base, while slow-decaying arousal allowed the agent to occasionally visit the center of the arena (Figure 3B). Competing dynamics between the two internal needs emerged when feeding the excitatory populations of our neural mass model with the corresponding homeostatic errors, allowing the dominant attractor to inhibit its opponent, thus resolving drive orchestration (Figure 3C). After carrying out 50 experiments, agent trajectories showed consistency in their occupancy pattern: a clear preference to visit the home base, navigate close to the walls, and avoid the center of the arena (Figure 3D). The distribution of the internal states during those 50 simulations was very informative. On the one hand, the agent maintained a high level of security during a large part of the simulations (Figure 3E). On the other hand, the state of arousal followed a bimodal distribution indicating the agent was either high or low aroused during the experiment. This bimodal distribution can be interpreted as follows: First, while the agent pursues security, an aversion toward the center of the arena induces low states of arousal. Then, low internal states, in turn, promote transversals that fully replenish the arousal of the agent (Figure 3F). Finally, attractor dominances, i.e., the simulation period where the firing rate of one excitatory neural pool exceeded the firing rate of the other, showed a balanced activation of both attractors with a slight bias toward arousal (Figure 3G).

### Dynamic environment

**FIGURE 4:**
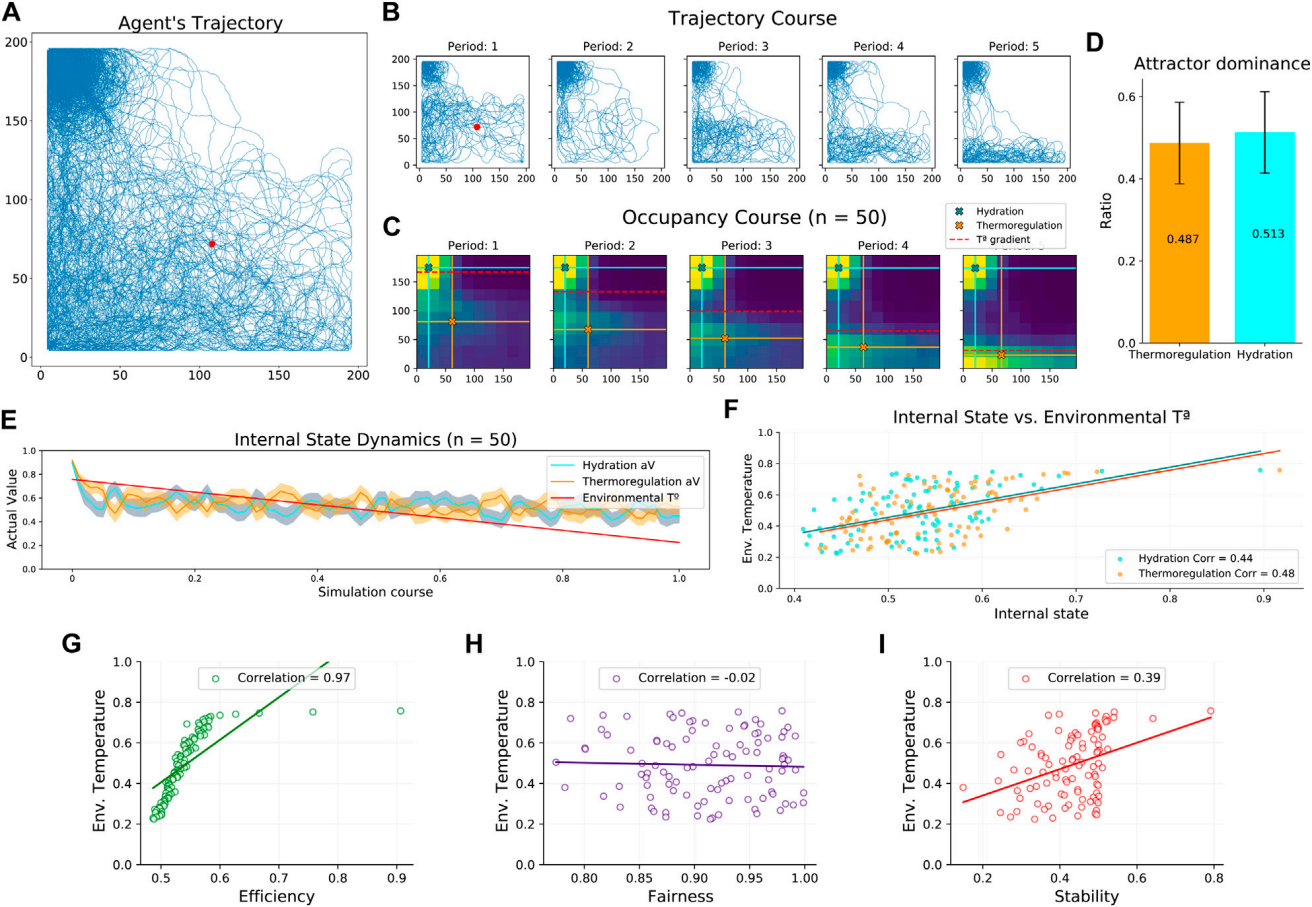
Agent’s performance in a dynamic environment. **(A)** Agent’s trajectory tracked along a complete experiment. The red dot represents starting location, randomized across experiments. **(B)** Agent’s trajectory tracked along a complete experiment (same as a) divided into five periods. **(C)** Map of occupancy divided into five periods. Color crosses illustrate the mean position during hydration (blue) and thermoregulation (orange) attractor dominance. The red dashed line illustrates the variable slope location of the dynamic temperature gradient. **(D)** Mean attractor dominance **(E)** Mean agent internal state dynamics and environmental temperature. The shaded area indicates the internal state variance. **(F)** Mean internal state dynamics correlated with the environmental temperature. **(G)** Efficiency dynamics correlated with the environmental temperature. **(H)** Fairness dynamics correlated with the environmental temperature. **(I)** Stability dynamics correlated with the environmental temperature. Aggregated results from 50 experiments.

In the second study, we assess the robustness of our novel neural mass allostatic model in a dynamic environment. In this condition, we observe that the agent’s trajectory accurately tracks the environmental gradients (Figure 2B) occupying their peak areas (Figure 4A, Supplementary Figure S6). Specifically, when dividing the complete experiment into five periods of equal length, we can observe that the agent’s trajectory constantly adapted to the changes in the temperature gradient (Figure 4B). Thus, when initializing the experiment (period 1), the peak area of the temperature gradient covers a large part of the arena, and the agent navigates more extensively. However, in the last period (period 5), the peak area is reduced to a thin region close to the bottom border, and the agent’s navigation adapts to it. This trajectory pattern was consistent across the 50 experiments, as the occupancy maps suggest (Figure 4C). Spearman correlation analysis indicated that the mean *Y* axis position of the agent during thermoregulation highly correlated with the temperature gradient’s slope location (*r*(498) = 0.96, *p* < 0.001), confirming the agent’s trajectory adaptation. Once again, attractor dominance was balanced (Figure 4D), suggesting that although the environment asymmetrically reduces the opportunities to fulfill the agent’s internal needs, the neural mass model imposes a well-balanced competition without neglecting any of the drives. Indeed, the mean internal state across the experiment shows that thermoregulation and hydration levels are well maintained without biases (Figure 4E), and both are equally correlated with the environmental temperature (mean value of the temperature gradient) (Figure 4F). To understand in detail the relationship between internal states and environmental dynamics, we studied this relationship in terms of efficiency, fairness, and stability metrics. Correlating these measures with the environmental temperature, we observed that efficiency strongly depends on the opportunities to fulfill internal needs (Figure 4G). This result was expected, given the environmental dynamics. When the environmental temperature decreases (i.e., temperature gradient peak shrinks), gradients’ peak areas are more distant, forcing the agent to navigate a larger area where its internal states decrease. However, environmental changes do not affect the fairness level (Figure 4H), validating that the balance of attractor dominance is defended even when receiving asymmetric influences from the environment. Lastly, stability reports difficulties minimizing the mean square homeostatic error as temperature decreases (Figure 4I). According to our previous analysis, this result would be better explained by environmental influences on efficiency than fairness.

### Criticality-driven decision-reset after decision accomplishment

**FIGURE 5:**
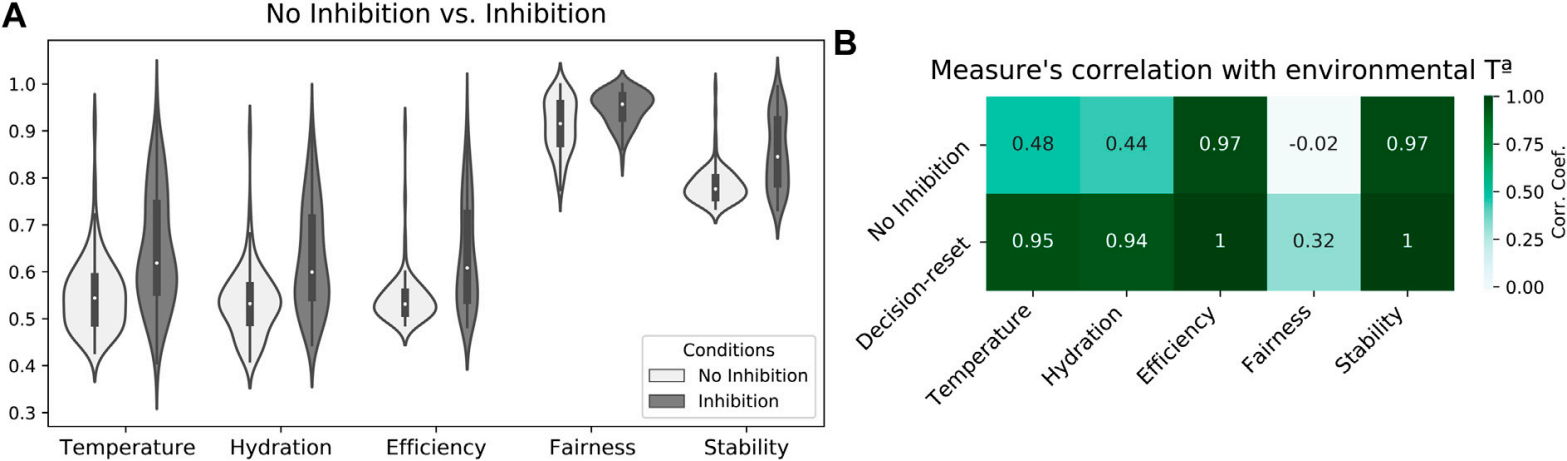
Decision-reset supports the agent’s internal stability. **(A)** Comparison of temperature and hydration internal states and efficiency, fairness, and stability scores between no-inhibition (study 2) and decision-reset (study 3) conditions. **(B)** Correlation between internal states and game theory measures with environmental temperature for both no inhibition and decision-reset conditions.

Finally, a third study explores the potential limitations of attractor-based allostatic models. Specifically, by inhibiting the model’s excitatory neural populations (once drive-completion behaviors have been performed), we explore whether sustained attractor forces could hamper individual self-regulation in study 2. Results showed that agents widely benefit from inducing a critical regime to set the initial conditions for each cycle of allostatic orchestration. Specifically, mean internal states along the simulation (calculated every 1/100 fraction of the experiment) across 50 simulations indicated that agents maintained their internal states better when applying decision-reset (Inhibition). (Figure 5A). Consequently, increased internal states also resulted in increased efficiency, fairness, and stability scores. A Mann-Whitney test indicated statistically significant differences when comparing the two conditions in each measure, *U*(*N*_*No*−*inhibition*_ = 100, *N*_*inhibition*_ = 100), *p* < 0.001). Furthermore, this enhanced performance occurred in larger alignment with the environmental temperature (Figure 5B), which indicates the agent’s internal states decreased when the scarcity of resources prevented a better self-regulatory strategy. Altogether, these results suggest that attractor forces sustained after drive-completion behaviors hampered allostatic orchestration, and criticality-driven decision-reset provides an effective mechanism to facilitate a more flexible decision-making process.

## Discussion

Previous computational works have contributed to explaining animal self-regulatory behavior (Sanchez-Fibla et al., 2010; Keramati and Gutkin, 2014; Jimenez-Rodriguez et al., 2020). However, these models have not been validated in a broad range of tasks with emphasis on their robustness in the face of varying task conditions, while in parallel, their grounding in the brain mechanisms underlying allostatic orchestration is not fully elucidated. We hypothesize that attractor dynamics originate from the competing relation between different hypothalamic interoceptive nuclei and their loops through the zona incerta, which are further perturbed by the superior colliculus (input and orienting control), and the central gray for triggering reactive species-specific behaviors. To test the adequacy of allostatic attractor dynamics in orchestrating internal needs, we built a biologically-constrained computational model of allostatic orchestration. The resultant model implements competition between internal drives by comprising two drive-sensitive excitatory neural populations that apply mutual and feedback inhibition. To allow a synthetic agent to navigate a virtual external environment based on its internal state, we endowed it with the ability to orientate and perform basic appetitive-aversive navigational behaviors. In this manner, the hypothalamus, superior colliculus, and zona incerta-periaqueductal gray axis are represented in our model, providing a first approximation of the core behavior system (Merker, 2013; Verschure, 2016).

Deploying the allocentric synthetic agent in a virtual environment, we tested the competencies and robustness of the neural mass allostatic model. Specifically, our model allowed the agent to 1) navigate an open field reproducing rodent behavior, 2) adapt its navigation to environmental changes without neglecting any internal needs, and 3) benefit from criticality reset to optimize the interoceptive-driven decision-making process. Altogether, our results supported our hypothesis empirically validating attractor dynamics as an inherent feature of the hypothalamic circuitry that can underlie robust allostatic orchestration. However, the attractor dynamics that emerge in our model need to be further validated by physiological data, ideally by direct neural recordings of the hypothalamic interoceptive nuclei. Lacking ground truth benchmarking data, the temporal dynamics of the homeostatic markers employed in this work were arbitrarily set, representing an additional methodological limitation. Moreover, now that we have assessed the robustness and fidelity of the model to track environmental dynamics, we need to assess its scalability.

Additionally, our work opens new questions in the quest to understand self-regulation. For instance, a better understanding of the interplay between stress and risky internal states is needed. In our studies, the level of competition between neural populations remained fixed at an optimal point. However, the ratio between mutual and shared inhibition could be modulated by stress markers such as acetylcholine (Kawaguchi, 1997). How stress can modulate the competition held between internal drives is a question that remains unanswered. Another open question is how allostatic orchestration interplays with predictive and contextual allostasis to assist each other. We propose to structure allostasis as a cognitive architecture following the four organizational layers of the Distributed Adaptive Control theory of mind (Verschure, 2012). Here, a first somatic level endows the agent with predefined internal needs. Then, the reactive level rapidly orchestrates behaviors directed to calm the most salient drives. In parallel, the adaptive layer learns associations between external and internal events to predict future homeostatic errors and solve them in anticipation. Lastly, a contextual layer enriches the representation of the self-regulatory strategies and enables their memorization.

Finally, contributions to the field of allostasis can expand the boundaries of self-regulation in its understanding and implementation. On the one hand, drives and motivations should also respond to a hierarchical organization that encodes not only priority but also abstraction, virtualization, and replaceability of the drive (Verschure, 2016). Maslow’s hierarchy was a first approximation to capture this organization (Maslow, 1943); however, 7 decades later, this theory has not been further advanced in the face of new insights into the dynamics of self-regulation. How different needs within the same priority level organize or how new needs arise and substitute previous ones are questions not answered yet. On the other hand, further investigation could shed light on how a hierarchical organization of needs fits with a neural mass model. Secondly, general-purpose robot instantiations can benefit greatly from the self-regulatory principles described here. Previous works elaborated on how a multi-agent robotic recycling plant can implement homeostatic and allostatic principles to self-organize at both single-agent and large-scale levels (Rosado and Verschure, 2020). Similar architectures can be designed and implemented to advance a variety of scenarios where robot autonomy is key such as robot delivery or space exploration. Indeed, autonomy in artificial intelligence and robotics places the question of self-regulation at the center of the research of synthetic embodied cognition and consciousness.

## Data Availability

The original contributions presented in the study are included in the article/Supplementary Material, further inquiries can be directed to the corresponding authors.
