# Supplementary material for: Drive competition underlies effective allostatic orchestration
Source: Front Robot AI. 2022 Dec 2;9:1052998. doi: 10.3389/frobt.2022.1052998 (PMC9755511; doi:10.3389/frobt.2022.1052998)
Supplement: Supplementary file 1 [file DataSheet1.PDF]

# Supplementary Material

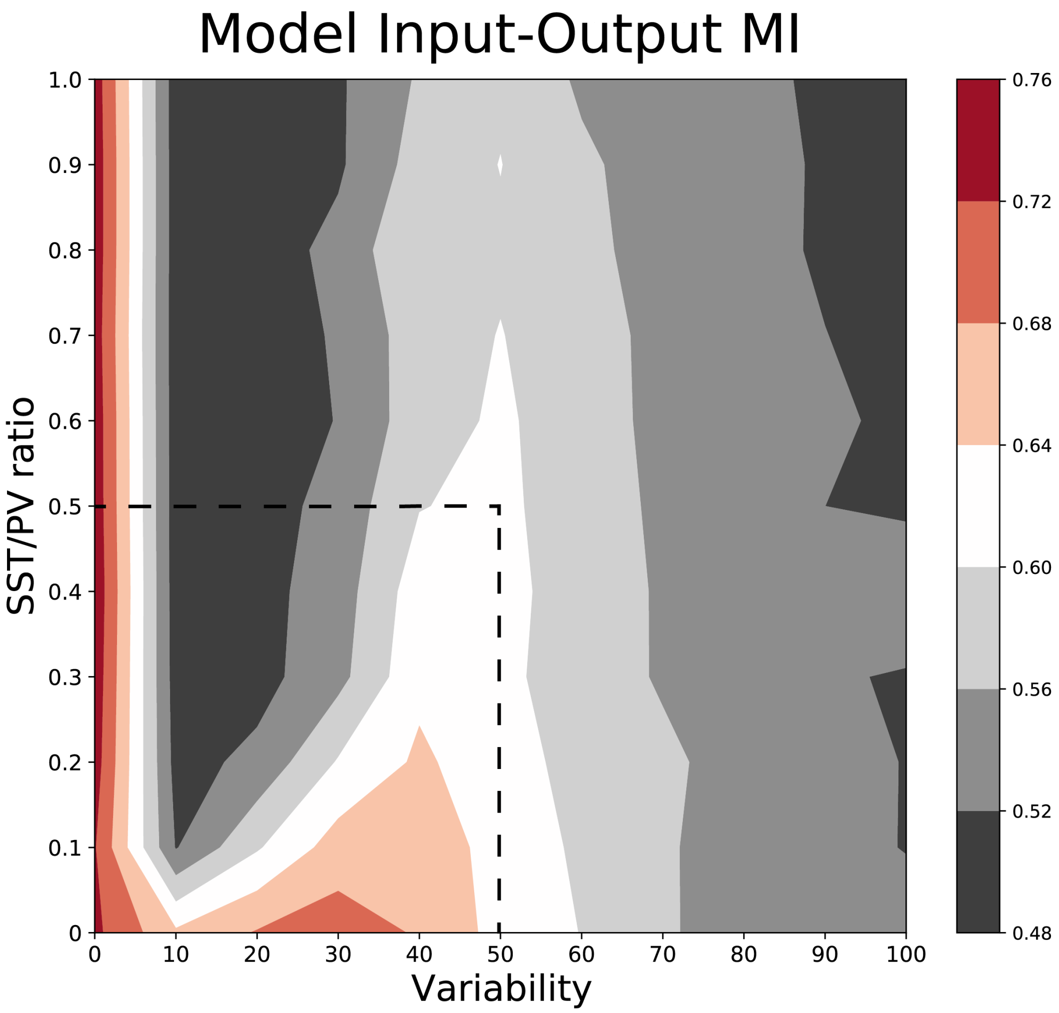


**Figure S1. SST/PV ratio and variability parameter search based on mutual information scores between artificial model inputs and outputs.** 11 levels of SST/PV ratio (STT: Mutual inhibition, PV: Feedback inhibition) and 11 levels of variability are combined to analyze the mutual information (MI) scores of attractor inputs and outputs. Each MI score is extracted from 10 experiments. Therefore, a total of 1.210 experiments (11 SST/PV ratios x 11 variability levels x 10 experiments) were carried out to perform the present figure. Importantly, the test is based on artificial inputs where first, arousal input is equal to 0, and security input increases linearly to 1 to decrease again to 0. Then, arousal input follows the same dynamics while security remains at 0. Finally, both drives increase linearly at a time to 1. The combination of 0.5 SST/PV ratio and 50 variability parameters provided high MI scores while maximizing the competing relation and the variability applied. Higher MI scores were found when parameters were set close to 0. We decided to rule out those combinations since it could be due to a low frequency of attractor switches and, in turn, to a bias in attractor dominance. See figures S2, S3, and S4 for more information.

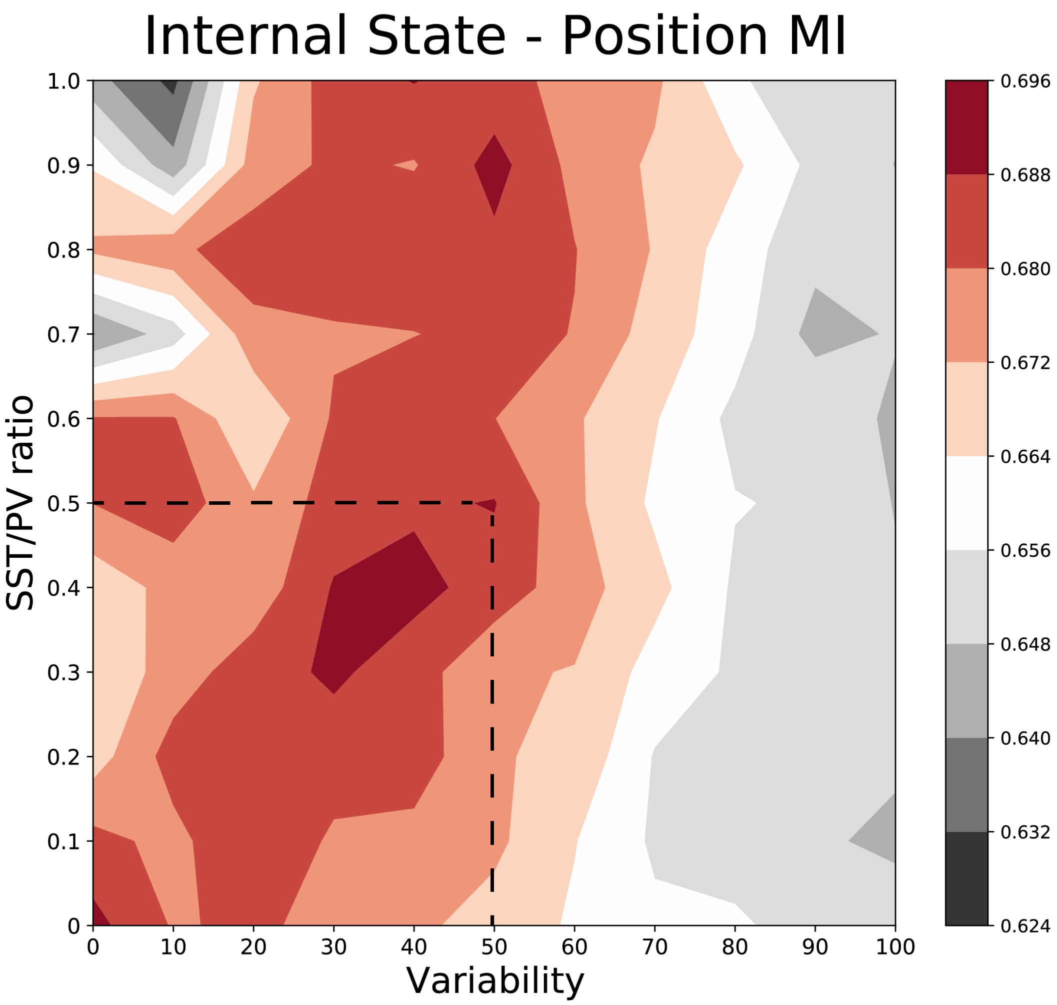


**Figure S2. SST/PV ratio and variability parameter search based on mutual information scores between agent internal state and its position during an open field test.** Mutual information analyses were carried out to double-check the selection of the SST/PV ratio and variability parameters, as in figure S1. In this case, the data used comes from 2.420 experiments (11 SST/PV ratios x 11 variability levels x 20 open field tests), and mutual information was based on the agent's internal states and its location in the arena along the experiment. Again, the combination of 0.5 SST/PV ratio and 50 variability parameters scored high in MI, reaffirming its suitability.

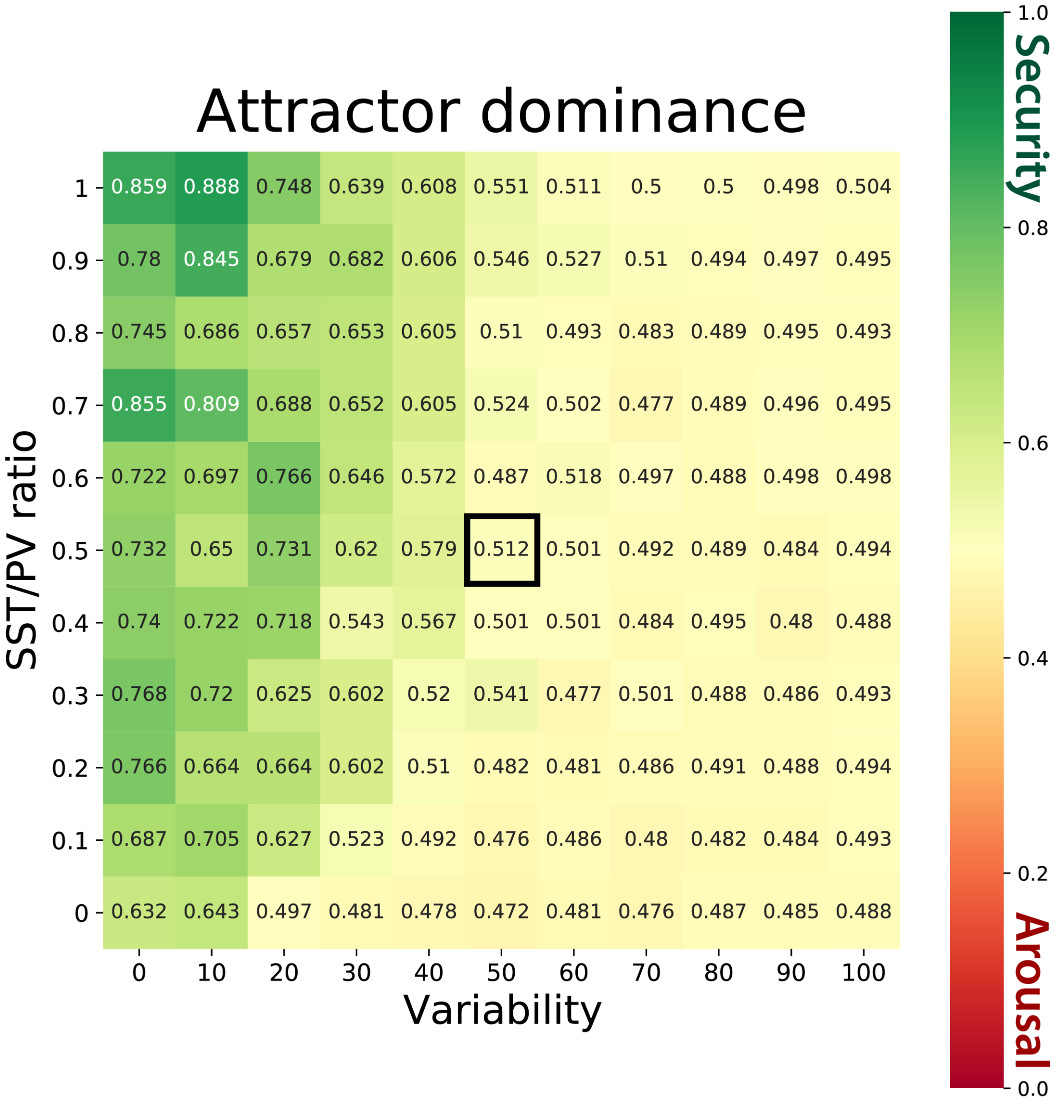


**Figure S3. SST/PV ratio and variability parameter search based on attractor dominance during an open field test.** Attractor dominance of the 2.420 open field tests carried out for figure S2 was analyzed to understand previous MI results better. As we foresaw in figure S1, low SST/PV ratio and variability values result in an attractor bias towards security, presumably due to a low prevalence of attractor switches (See figure S4 for more information). This bias always occurred in favor of security. This effect is explained by the faster temporal dynamics of security, which feeds the model with a higher homeostatic error in the early stages of the experiments. Thus, the models enter the security attractor state and struggle to escape it.

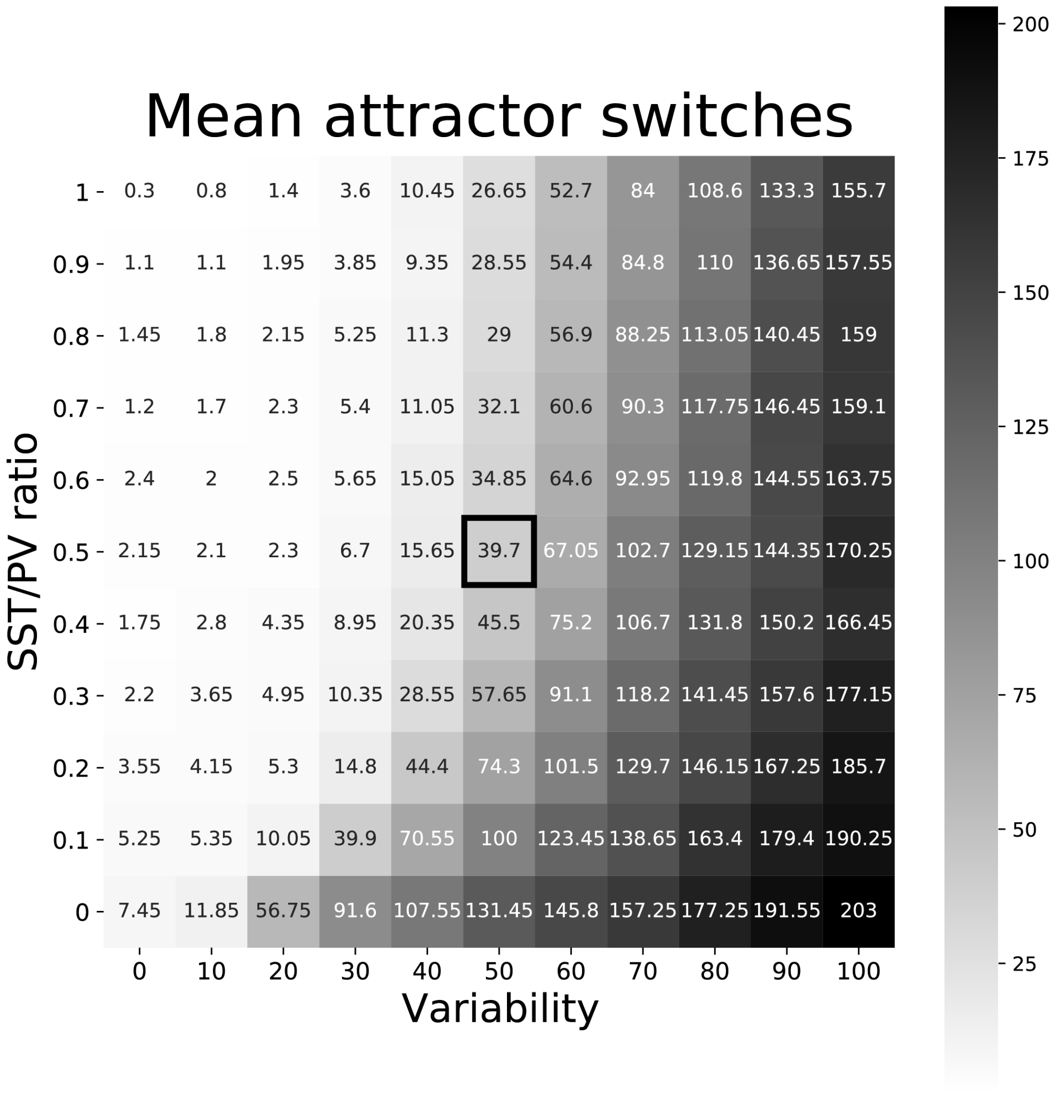


**Figure S4. SST/PV ratio and variability parameter search based on attractor switches during an open field test.** Mean attractor switches of the 2.420 open field tests carried out for figures S2 and S3 were analyzed to understand previous MI and attractor dominance results better. As we foresaw in figure S1, low SST/PV ratio and variability values result in a low prevalence of attractor switching, which could explain the biases in attractor dominance.

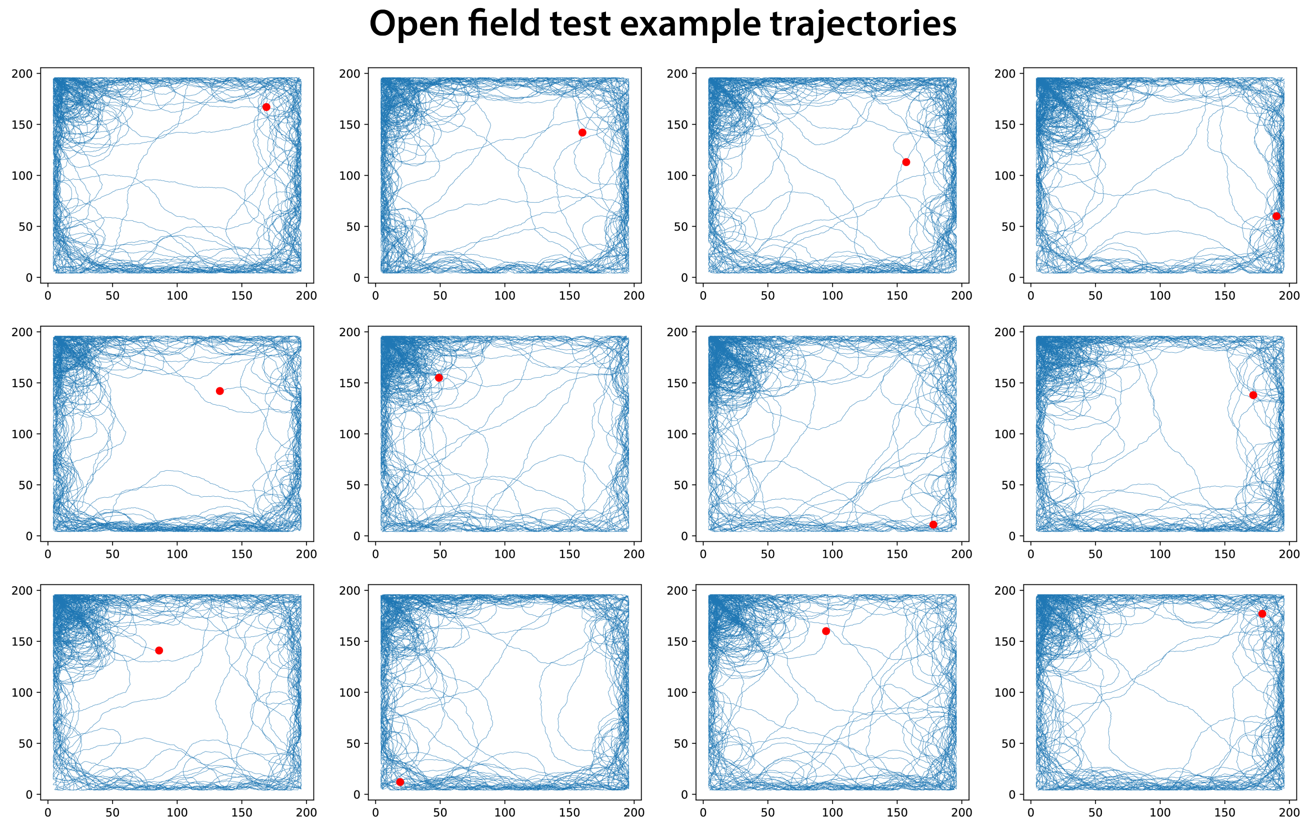


**Figure S5. Open field test trajectory examples.** 12 example trajectories out of the 50 carried out open field tests. The red dot illustrates the randomized starting position of the agent. Due to the system's complexity, specific trajectories are impossible to predict. However, the agent's navigational pattern persists across experiments.

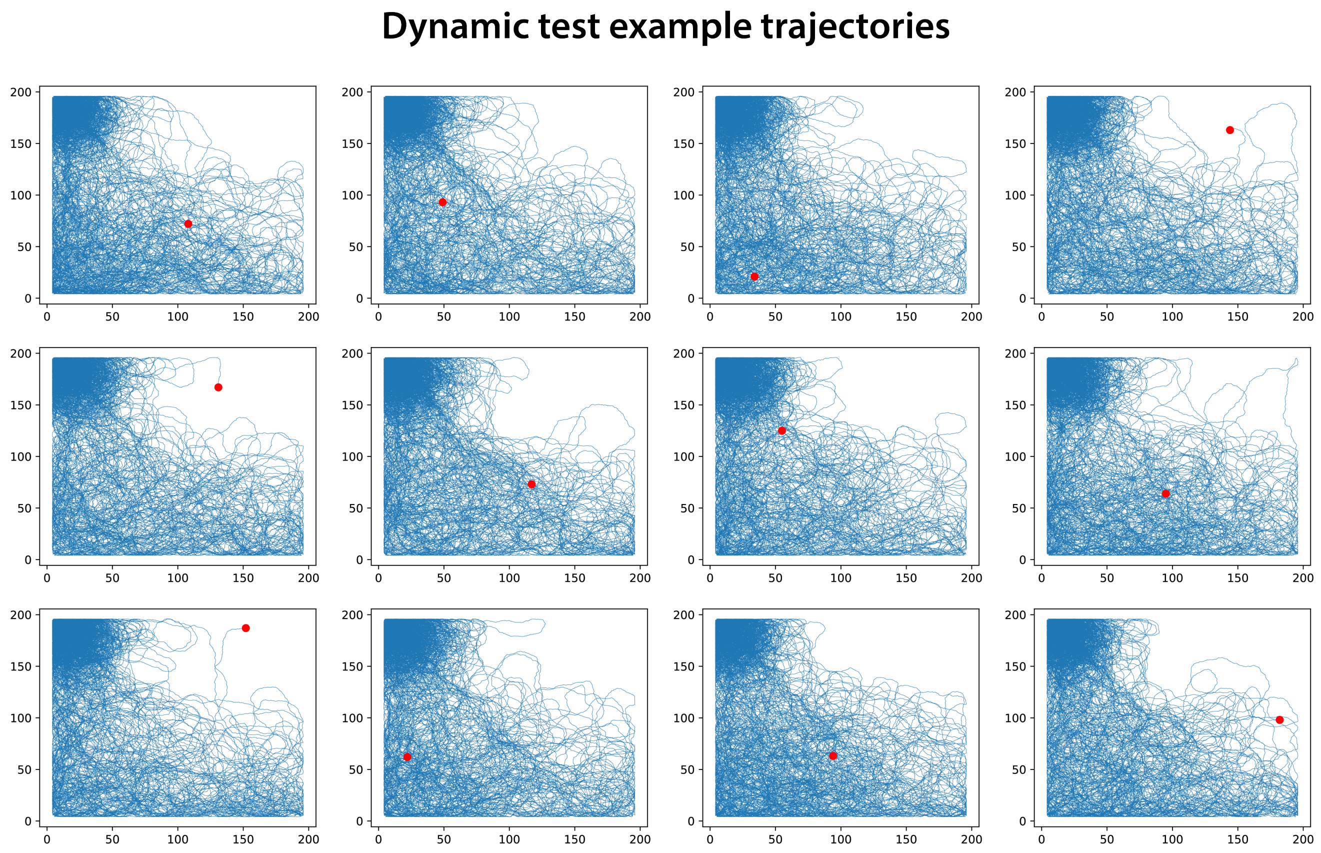


**Figure S6. Dynamic test trajectory examples.** 12 example trajectories out of the 50 carried out dynamic tests. The red dot illustrates the randomized starting position of the agent. Due to the system's complexity, specific trajectories are impossible to predict. However, the agent's navigational pattern persists across experiments.
